# College Announcements

**Published:** 1858-10

**Authors:** 


### COLLEGE ANNOUNCEMENTS.

In addition to the large number of documents of this description received and noticed heretofore, we find upon our table the "Annual Announcement of the Savannah Medical College for the Session of 1858–9, from which we learn that "The Sixth Session of the Institution will commence on the first Monday in November next.

Preliminary Lectures, which will be free, will commence on the 18th of October.

The Faculty, encouraged by the liberality of the last Legislature to the College, will enter upon their annual duties with renewed zeal and energy."

We have also received the "Annual Circular of the Memphis Medical College," the regular course of Lectures, in which, will commence on the 1st day of November 1858, and continue until the first of March. We learn from the Announcement that the anticipations of the Faculty, as expressed upon previous occasions, have been completely fulfilled and that the Institution, during the last seven years, has made many decisive steps towards perfection. Possessing one of the most favorable points in tne Southern country for a Medical School, it should be eminently successful. In this connection we would correct an error in the last number of our Journal in the notice of the Oglethorpe Medical College of Savannah,—the number of Matriculates was 37, instead of 30, as stated.

